# Safety aspects of the PiCCO thermodilution-cardiac output catheter during magnetic resonance imaging at 3 Tesla

**DOI:** 10.1007/s10877-020-00630-8

**Published:** 2021-01-05

**Authors:** Marieke Voet, Christiaan G. Overduin, Ernst L. Stille, Jurgen J. Fütterer, Joris Lemson

**Affiliations:** 1 Department of Anesthesiology, Pain and Palliative Medicine, Radboud University Medical Center, P.O. Box 9101, HP 717, 6500 HB Nijmegen, The Netherlands; 2 Department of Radiology and Nuclear Medicine, Radboud University Medical Center, Nijmegen, The Netherlands; 3 Department of Intensive Care Medicine, Radboud University Medical Center, Nijmegen, The Netherlands

**Keywords:** MRI, MRI safety, Thermodilution, Cardiac output monitoring, Intravascular catheter, PiCCO™

## Abstract

Thermodilution cardiac output monitoring, using a thermistor-tipped intravascular catheter, is used in critically ill patients to guide hemodynamic therapy. Often, these patients also need magnetic resonance imaging (MRI) for diagnostic or prognostic reasons. As thermodilution catheters contain metal, they are considered MRI-unsafe and advised to be removed prior to investigation. However, removal and replacement of the catheter carries risks of bleeding, perforation and infection. This research is an in vitro safety assessment of the PiCCO™ thermodilution catheter during 3 T Magnetic Resonance Imaging (3T-MRI).  In a 3T-MRI environment, three different PiCCO™ catheter sizes were investigated in an agarose-gel, tissue mimicking phantom. Two temperature probes measured radiofrequency-induced heating; one at the catheter tip and one at a reference point. Magnetically induced catheter dislocation was assessed by visual observation as well as by analysis of the tomographic images. For all tested catheters, the highest measured temperature increase was 0.2 °C at the center of the bore and 0.3 °C under “worst-case” setting for the tested MRI pulse sequences. No magnetically induced catheter displacements were observed. Under the tested circumstances, no heating or dislocation of the PiCCO™ catheter was observed in a tissue mimicking phantom during 3T-MRI. Leaving the catheter in the critically ill patient during MRI investigation might pose a lower risk of complications than catheter removal and replacement.

## Introduction

Cardiac output monitoring is frequently used to analyze the hemodynamic condition and guide therapy in critically ill patients. The clinical gold standard cardiac output monitor is based on the thermodilution technique, which requires a thermistor-tipped intravascular catheter. As these catheters all contain ferro-magnetic material, they are considered unsafe in patients requiring Magnetic Resonance Imaging investigation (MRI). [1, 2]

A frequently used cardiac output monitoring system is the PiCCO™ device (Getinge group, Germany). It has demonstrated comparable accuracy and less complications than the pulmonary artery catheter and has the advantage to be validated in children.[3, 4] It requires a standard central venous catheter and a (thermistor-tipped; PiCCO™) catheter placed in a large artery. This may become a safety hazard when critically ill patients with a PiCCO™ catheter need an MRI for diagnostic or prognostic reasons. The magnitude of this hazard is defined by the magnetic field strength and radiofrequency (RF-)power. Higher field strengths induce more energy, increasing the risk of heating and dislocation of ferro-magnetic material. In clinical practice, imaging at higher field strengths, like 3 Tesla (T), is increasingly used because of their superior image quality. This might increase the safety hazard, but temporary removal and replacement of the catheter imposes other risks, like bleeding, infection or vascular perforation. Therefore, patients with a PiCCO™ catheter often are refrained from having an MRI.

Two reports describing the safety risk investigation of PiCCO™ catheters, found no clinically relevant heating or dislocation in a 1.5T MRI setting.[5, 6]. However, conclusions about MRI safety can only be drawn for the specific conditions and environment tested. Therefore, we wanted to investigate the safety hazards of our patients with an indwelling PiCCO™ catheter during 3T-MRI. Hence, we studied RF-induced heating and magnetically induced dislocation of three different PiCCO™ catheters in a tissue-mimicking phantom during 3T-MRI.

## Materials and methods

A risk assessment was performed to determine the safety aspects of the PiCCO™ thermodilution catheter in a 3T-MRI suite by measuring dislocation and heating of the catheters. Magnetically induced catheter movement or dislodgement was assessed by visual observation upon introduction to the magnetic field as well as by analysis of the tomographic images. Heating effects were assessed following the American Society for Testing of Materials (ASTM) standard test method for measurement of RF-induced heating during MRI (F2182-11).[7].

A tissue mimicking phantom (32 × 40 × 10 cm) was prepared of 30 g L^− 1^ agarose gel and 2 g L^− 1^ sodiumchloride to obtain tissue equivalent dielectric properties. The phantom was placed in a 3T clinical MRI system (Magnetom Skyra, Siemens, Erlangen, Germany) with a body phased-array coil placed over the phantom for imaging. Three commercially available sizes of the PiCCO™ catheter were tested: the 5F/20 cm (PV2015L20-A), 4F/16 cm (PV2014L16-A) and 3F/7 cm (PV2013L07-A) (PULSION, Getinge group, Germany). A fiberoptic temperature probe (T1, Neoptix, Quebec, Canada) was secured to the tip of each catheter and the catheters were fully inserted into the phantom with only the connector and one cm of the distal catheter remaining outside the phantom. Another temperature probe was placed at the contralateral side of the phantom for temperature reference.

**Fig. 1:**
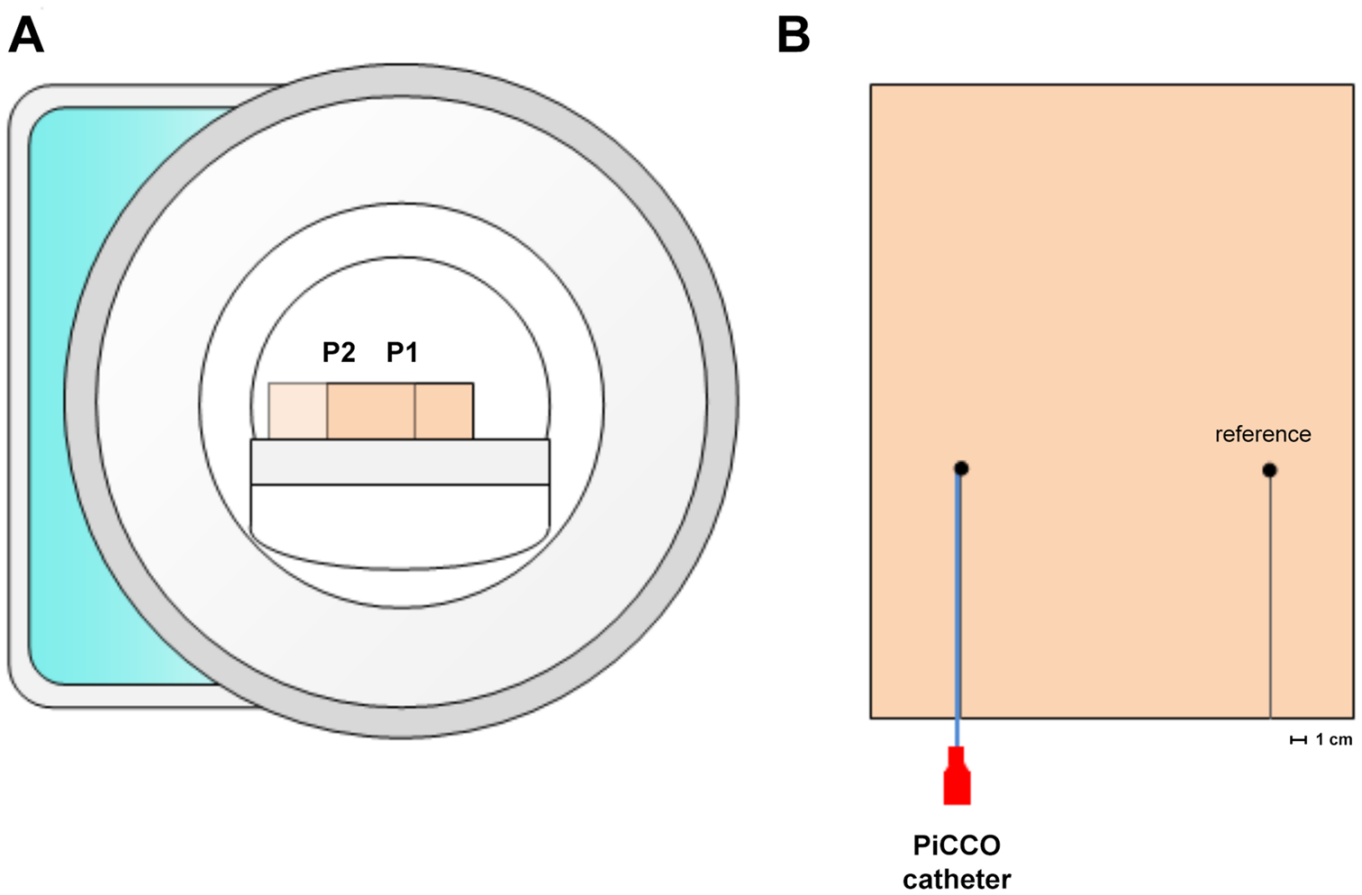
Schematic overview of the experimental setup for the RF-induced heating experiments. **a** position of the phantom in the MRI. Temperature measurements at two catheter positions within the scanner bore: P1 (center) and P2 (maximum off-center). **b** position of the catheter and temperature probes in the phantom. Left; fully inserted PiCCO™ catheter with fiberoptic temperature probe (●). Right; reference temperature probe on contralateral side of the phantom (●)

A schematic overview of the experimental setup is shown in Fig. 1; a shows the position of the phantom in the MRI, with P1 and P2 representing temperature probe positions in the scanner bore. b shows the position of the catheter and temperature probes in the phantom.

Measurements were performed at two locations within the scanner bore: (P1) center of the bore, representing the normal clinical setting; (P2) maximum off-center position close to the side of the bore, representing a worst-case setting since local electric field components and thus potential heating effects are highest closer to the edge of the bore. MRI was performed using four clinical routine MR pulse sequences (T1 VIBE, T2 TSE, T2 HASTE, TRUFI), one sequence that is evaluated in a scientific setting (pcASL) and one sequence that was modified to produce the maximum allowed RF-power within the specific absorption rate limits of the scanner (Modified TRUFI). The last sequence represents the worst-case condition for energy deposition. A detailed overview of the tested sequence types and parameters is displayed in Table 1.

**Table 1:** Overview of the tested sequence parameters for MR imaging

Setting	Name	Sequence type	TR/TE (ms)	Flip angle (°)	Total slices	Slice thickness (mm)	Field of view (mm^2^)	Matrix size	Imaging plane	Acq. time (min)	WB-SAR (% of 4 W/kg)	Time-averaged RF power (W)
Clinical routine	T1 VIBE	Spoiled gradient echo	4.4/1.9	6	40	2.5	300 × 300	192 × 192	Coronal	1:30	12	8.3
	T2 TSE	Turbo spin echo	4490/110	150	31	3.0	160 × 160	256 × 230	Coronal	1:30	60	41.3
	T2 HASTE	Half fourier single-shot turbo spin echo	3000/102	180	29	5.0	300 × 300	256 × 256	Coronal	1:30	66	45.2
	TRUFI	Balanced steady state free precession	4.3/2.1	65	16	3.0	280 × 280	256 × 256	Coronal	1:30	98	68.1
Scientific setting	pcASL	Turbo gradient spin echo	5000/19	180	16	5.0	300 × 150	64 × 32	Coronal	5:05	46	38.4
Maximum RF-power	Modified TRUFI	Balanced steady state free precession	4.3/2.1	49	5	3.0	280 × 280	256 × 256	Coronal	1:30	100	95.2

*TR/TE* Repetition Time/Echo Time, *WB-SAR* Whole-Body averaged Specific Absorption Rate, *RF* Radio Frequency, *VIBE* Volumetric Interpolated Breath-hold Examination, *TSE* Turbo Spin Echo, *HASTE* Half-fourier Acquisition Single-shot Turbo spin Echo, *TRUFI* True Fast Imaging with steady-state free precession, *pcASL* pseudo-continuous Arterial Spin Labeling, *modified TRUFI* worst-case condition energy deposition

Each measurement was repeated at least three times for each catheter size and location within the scanner bore. Temperature changes throughout the experiments were recorded at 1 second intervals. Maximum temperature increases (ΔT_max_) are presented as mean (SD)[range].

## Results

Baseline temperature of the phantom was 18.5 (± 0.9)°C for all but one pulse sequences. The sequence for scientific setting was tested in a separate session, with a baseline temperature of 21.2 (± 0.4)°C. The maximum temperature changes recorded at the PiCCO™ catheter tip during scanning are shown per catheter size and measurement location in Table 2. For all tested catheters and pulse sequences, the highest measured temperature increase was 0.2 °C at the center of the bore and 0.3 °C under off-center conditions.

**Table 2:** Maximum temperature changes at each catheter tip, for each sequence during 3T-MRI

MRI sequence	PiCCO catheter size			
	5F/20 cm	4F/16 cm	3F/7 cm	Reference
	ΔT_max_ (°C)	ΔT_max_ (°C)	ΔT_max_ (°C)	ΔT_max_ (°C)
Center of the scanner bore				
T1 VIBE	**0.1** (0.0) [0.0-0.1]	**0.1** (0.0) [0.0-0.1]	**0.0** (0.0) [0.0–0.0]	**0.1** (0.0) [0.0-0.1]
T2 TSE	**0.1** (0.0) [0.1–0.1]	**0.1** (0.0) [0.1–0.1]	**0.1** (0.0) [0.0-0.1]	**0.1** (0.0) [0.0-0.1]
T2 HASTE	**0.1** (0.0) [0.1–0.1]	**0.1** (0.0) [0.1–0.1]	**0.1** (0.0) [0.0-0.1]	**0.1** (0.0) [0.0-0.1]
TRUFI	**0.1** (0.0) [0.0-0.1]	**0.1** (0.0) [0.0-0.1]	**0.1** (0.0) [0.0-0.1]	**0.1** (0.0) [0.0-0.1]
pcASL	0.1 (0.0) [0.1–0.1]	**0.1** (0.0) [0.0-0.1]	**0.1** (0.0) [0.0-0.1]	0.1 (0.0) [0.0–0.0]
Mod. TRUFI	**0.2** (0.1) [0.0-0.2]	**0.2** (0.1) [0.0-0.2]	**0.1** (0.0) [0.0-0.1]	**0.1** (0.0) [0.0-0.1]
Off-center position				
T1 VIBE	**0.1** (0.0) [0.1–0.1]	**0.0** (0.0) [0.0–0.0]	**0.0** (0.0) [0.0–0.0]	**0.1** (0.0) [0.1–0.1]
T2 TSE	**0.3** (0.1) [0.1–0.3]	**0.2** (0.1) [0.0-0.2]	**0.1** (0.0) [0.0-0.1]	**0.1** (0.0) [0.1–0.1]
T2 HASTE	**0.1** (0.0) [0.1–0.1]	**0.1** (0.0) [0.1–0.1]	**0.1** (0.0) [0.1–0.1]	**0.3** (0.1) [0.0-0.3]
TRUFI	**0.2** (0.0) [0.1–0.2]	**0.1** (0.0) [0.1–0.1]	**0.1** (0.0) [0.0-0.1]	**0.3** (0.1) [0.1–0.3]
pcASL	**0.2** (0.1) [0.1–0.3]	**0.1** (0.1) [0.0-0.2]	**0.1** (0.0) [0.1–0.2]	0.2 (0.1) [0.1–0.3]
Mod. TRUFI	**0.2** (0.1) [0.0-0.2]	**0.2** (0.1) [0.0-0.2]	**0.1** (0.0) [0.0-0.1]	**0.1** (0.0) [0.1–0.1]

Results are presented as mean(SD)[range]. clinical routine MR pulse sequences; *VIBE* Volumetric Interpolated Breath-hold Examination, *TSE* Turbo Spin Echo, *HASTE* Half-fourier Acquisition Single-shot Turbo spin Echo, *TRUFI* True Fast Imaging with steady-state free precession, *pcASL* pseudo-continuous Arterial Spin Labeling, *modified TRUFI* worst-case condition energy deposition

None of the experiments showed magnetically induced movement or accidental dislodgement of any of the catheters, observed visually and in the acquired MR images.

## Discussion

Three different PiCCO™ catheter sizes were tested for RF-induced heating and magnetically induced dislocation during 3T-MRI in a phantom. None of the tested PiCCO™ catheters showed clinically significant heating or dislocation even in extreme RF-conditions. These findings are in line with earlier reports on testing in a 1.5T-MRI, where no dislocation or clinically significant heating was found [5, 6].

Our in vitro-model is comparable to the human body concerning volume, and thermal and electrical properties. Although electrical parameters are temperature dependent following a linear relationship in the range of interest (20–100 °C), irreversible changes are only reported to occur at temperature extremes (> 80°C), which did not occur during our measurements [8]. We therefore consider the temperature-dependent behavior of the in-vitro model comparable to that of biological tissue. Although the model’s baseline temperature was less than body temperature, significant differences in induced current or heat are not expected as a function of baseline temperature, in relation to the effect of the temperature change itself. Therefore, the resultant relative temperature changes are considered similar to those that would occur in human tissue at body temperature.

Implants and devices considered MRI-unsafe under the classification are advised to be removed before a patient enters the MRI suite to prevent excessive heating and burns [1]. Most reports on MRI-related patient burns can be explained by the resonant circuit or antenna effect of monitoring cables [9]. Especially sedated patients, e.g. critically ill patients, are at risk as they cannot report any heating or discomfort due to device heating or dislodgement during scanning. Therefore, it is advised to remove a hemodynamic monitoring catheter before MRI scanning. However, removal and replacement of the catheter might pose a larger risk to the patient’s clinical condition than the presence of the catheter during MRI.

Although in vitro tests suggest a good safety margin for these catheters in an MRI environment, definite conclusions can only be drawn for the tested circumstances. Variables as catheter length, configuration and orientation of the metal wire, isolation material in the device and catheter positioning in air or body all interact and influence RF-induced heating. Consequently, translation of these test results should be evaluated in the specific clinical situation and discussed with local MR safety authorities. When deciding to keep the catheter during MRI, precautions have to be taken. Cables attached to the PiCCO™ catheter can produce heating or dislocation caused by induction of an electrical current, especially when in loops, so they should be removed before entering the MRI suite. Also, the metal inset of the connector should not be exposed nor be in direct contact with the patient’s skin and should be fixated to prevent accidental dislocation during scanning.

*In conclusion*, no considerable hazard due to RF-induced heating or dislocation of the three commercially available PiCCO™ catheter sizes were observed during 3T-MRI under the tested circumstances. So, leaving the catheter in the critically ill patient during MRI investigation could be considered if it is expected to pose a lower risk of complications than catheter removal. However, in collaboration with the local MR authorities, specific precautions have to be taken. Ultimately, one could consider an *in vitro* experiment in the own MRI environment to optimally asses patient safety.

## Data Availability

All data are available in the presented tables.
